# Hay-Fever

**Published:** 1887-07-30

**Authors:** 


### The Doctor's Pulpit.

### HAY-FEVER.

Several years ago a metropolitan doctor in search of sea-air went down to Scotland by water. On board the vessel was an underwriter, with whom the doctor entered freely into conversation. So long as the boat was in the Thames, the underwriter seemed to be in the best of health and spirits, but when the open sea was fairly reached he began to snuffle, look red about the eyes, and to use his pocket-handkerchief almost constantly. As night came on he grew worse and worse, until the doctor, who had a sympathetic soul, longed to prescribe for him, even without fee. Physic, however, there was none on board: and if there had been a whole chemist's shop, the probability is that no drug would have checked the attack. The patient was a philosopher. He said quite calmly, almost indifferently : "This is exceedingly inconvenient, but I always have it when on board ship in the open sea ; it will leave me in an hour when I get on shore." For thirty-six hours the sneezing continued almost without interruption ; the patient used up all his clean pocket-handkerchiefs, and his nose and eyes were red and swollen with acute catarrh. Within an hour of his landing at Leith he was quite well ; no discharge, no discomfort, no need for fresh pocket-handkerchiefs. The cure was complete. Mr. —— called his attack "hay-fever," and said it was in every respect similar to attacks of genuine hay-fever, to which he was subject when he was exposed to the pollen of hay. Would Dr. Precision call it by some other name ?

What was the cause of the attack ? What was the *modus operandi* of the cure ? The cause apparently was not the chill air of the sea, as some people might suppose, because the cure was immediate, although the sufferer landed at Leith in the cold night after being all day under a blazing sun. During the heat of the day, whilst he was on the water, he remained bad ; during the cold of an Edinburgh evening, when he was on land, he was quite well. Apparently, then, the cause was to be found in something which pervaded the atmosphere of the sea. What was it ? It might have been the particles of common salt with which the air was impregnated, or it might have been the iodine which is present in small quantities in sea-air. Probably it was the iodine, because every doctor knows that in some people very minute quantities of iodine, either inhaled or swallowed, will bring on symptoms similar to those of hay-fever almost immediately.

But iodine has nothing to do with a hay-field ! Certainly not. Is it not possible that there may be things in a hay-field which will produce a similar effect to iodine ? Iodine is a powerful irritant : so is snuff: so is ammonia: so are many things. There are irritating particles in hay-fields which have the power of setting up hay-fever. Does this condition occur nowhere except in hay-fields or on the sea ? As a matter of fact it very seldom occurs on the sea. A short voyage, indeed, is often the promptest cure. It may occur anywhere,—miles from a hay-field and leagues from the sea. Given the right man, with certain conditions of atmosphere, and you have a match at a powder-magazine. Some men get attacks of hay-fever on the slightest provocation ; other men can hold their noses over a bushel of snuff almost without sneezing. They can go to sea in the coldest weather, lie on deck all night, or even face the regions of the frozen North, without so much as a single cold in the head. Hay-fever depends almost entirely on the person, and hardly at all, for ordinary people, on the temperature, or the place, or the peculiar condition of the atmosphere.

What can the doctor do for it ? Something ; but very little. What can the patient do? Very much. As a rule he can cure himself. He has taken the fever in a certain atmosphere: let him find out an exactly opposite atmosphere. Has he contracted the attack at the seaside? Then let him seek mountain-air. Has the cold air of the hills brought it on? Then he must find his way to the milder atmosphere of the plains. Is it pollen or other powder incidental to hay-fields that is the offender? Then he must eschew hay-fields until the corn is yellow and the hay-harvest past and done with. Whether the disease be the result of merely local irritation of the mucous membrane of the air-passages, or of the physiological action of an inhaled atmospheric constituent which has been taken into the general circulation, it is quite impossible to determine. We know that iodide of potassium taken into the stomach will produce naso-laryngeal catarrh as promptly as when inhaled by the nostril. It cannot therefore be concluded with certainty that local irritation is the exciting cause. The probability is all the other way, and points to a constitutional rather than a local *raison d'être*. The discussion of this point may be left to experts; and as hay-fever is not a very deadly affair, it is not probable that any English or French Pasteur will commence a series of experiments for the elucidation of its nature and treatment.

The practical points connected with it are two: cure and prevention. As we have shown, the cure is not to be effected homœopathically, but by contraries. If homœopathy were a sound principle, the underwriter who got hay-fever by being on the sea should have been cured by going further on the sea; whereas he was cured, and that promptly, by transferring himself to dry land. And similarly, iodide of potassium, which, it is well known, will produce a naso-laryngeal catarrh which cannot be distinguished from hay-fever, ought, if homœopathy were true, to be an absolute specific for the disease. But as a matter of fact it only makes it worse. A total change of the atmospheric conditions in which the fever was developed is the quickest and most effective method of cure.

Sir Andrew Clark, who confesses he has tried almost everything, speaks with some hope of a plan, the principle of which is to exhaust the reactionary power of the nasal mucous membrane. He at the same time improves the general health. He uses this mixture: an ounce of glycerine of carbolic acid, a drachm of hydrochlcrate of quinine, and a two thousandth part of perchloride of mercury. The method is as follows: The nostrils are cleansed with a warm douche of boroglyceride and water—an ounce to a pint. A laryngeal brush is then dipped into the carbolic mixture until it is full, but not too full. The nostrils are carefully held open, and the brush gently but firmly pushed into each nostril as far as possible without injury, the brush being moved about so as to be brought into contact with every part of the nostril. Great care is to be taken to prevent the carbolic mixture touching or remaining in contact with any of the adjacent and non-affected parts. This treatment is often decidedly disagreeable, but Sir Andrew says it has been successful in a considerable number of cases, and that it may be recommended with some confidence for certain patients.

But prudent people who have had one or two attacks soon learn to avoid danger. Have they suffered from sitting at work under an open window? They will not again have the window open so close to their heads, even though the heat should be stifling. Does a trip to Scotland by sea bring on an attack? They will go by the Great Northern, the Midland, or the London and North-Western next time. Is the ripe grass or the new-mown hay the enemy? They will stick to town in July, and wait for their holidays until August or September if need be. It is wonderful what a little foresight and self-denial can do in the prevention of this most annoying disease. "A man is either a fool or a physician at forty," is one of those sayings which contains but a modicum of truth; but in the prevention of such diseases as hay-fever, bronchitis, and rheumatism, a man should exercise that kind of medical intelligence which is easily gained by one or two experiences, and which will constitute him his own physician to a certain limited extent. An open eye and a firm will can keep some diseases at bay as well as many other evils.

*(To be continued.)*

